# Systematic bioinformatics analysis reveals the role of shikonin in blocking colon cancer progression by identifying senescence-induced genes

**DOI:** 10.3389/fphar.2024.1360587

**Published:** 2024-08-12

**Authors:** Wenna Liu, Yujia Zhao, Qingqing Liu, Dan Wu, Wenxuan Li, Zhenkai Fu, Le Yang, Ying Liang

**Affiliations:** ^1^ Precision Pharmacy and Drug Development Center, Department of Pharmacy, Tangdu Hospital, Air Force Medical University, Xi’an, Shaanxi, China; ^2^ Department of Oncology, The First Affiliated Hospital, School of Medicine, Xi’an Jiaotong University, Xi’an, Shaanxi, China; ^3^ Laboratory of RNA Epigenetics, Institutes of Biomedical Sciences, Shanghai Medical College, Fudan University, Shanghai, China; ^4^ School of Basic Medical Sciences, Peking University, Beijing, China

**Keywords:** shikonin, cell senescence, bioinformatics, antitumor, molecular docking

## Abstract

Shikonin, a naturally occurring naphthoquinone compound extracted from comfrey plants, has antitumor, anti-inflammatory, and antimicrobial properties. Cell senescence plays a key role in preventing tumor progression. It is unclear whether shikonin has an effect on cell senescence in colon cancer. In the current study, we first determine the IC_50_ values of shikonin on colon cancer cell lines HT29 and HCT116. Then, we verified the inhibitory effects of shikonin on the proliferation and migration abilities of colon cancer cell lines HT29 and HCT116 using cell counting kit-8, colony formation, and wound healing assays. Next, we identified a series of potential targets using high-throughput mRNA sequencing and identified 210 upregulated and 296 downregulated genes. KEGG profiling revealed eight downregulated genes associated with cell senescence: *CCNB3*, *IL-1α*, *CXCL8*, *CDKN2A*, *MYC*, *IGFBP3*, *SQSTM1*, and *GADD45G*. Among them, *CXCL8* and *CDKN2A* were associated with poor prognosis in patients with colon cancer, suggesting that their downregulation by shikonin could improve patient survival. Furthermore, SA-β-galactosidase staining revealed that the percentage of cellular senescence in colon cancer cells was significantly increased after shikonin treatment. Molecular docking revealed that shikonin suppressed colon cancer progression by blocking *CXCL8* activity. Based on these findings, we deem that shikonin might induce senescence and exert antitumor activity in colon cancer cells by downregulating *CDKN2A* and *CXCL8*. This provides a new molecular mechanism and potential therapeutic target for shikonin to inhibit colon cancer progression.

## 1 Introduction

Colon cancer is the third most common malignancy worldwide with high morbidity and mortality (Shaukat et al., 2021). The inducement of colon cancer is multifaceted, encompassing various factors such as age, prolonged unhealthy dietary habits, and lifestyle choices, all of which frequently contribute to tumorigenesis. This complex interplay has resulted in a notable rise in colon cancer cases, with a particularly significant increase in the proportion of younger patients (DeDecker et al., 2021). Currently, therapeutic choices such as colon resection, radiotherapy, and chemotherapy are employed to arrest the progression of these malignancies. However, due to the absence of more sensitive screening techniques, surgical intervention is not feasible for some patients. Furthermore, the excision of the colon may cause substantial physical damage. Additionally, a considerable number of patients undergoing chemotherapy and radiotherapy experience recurrences, drug resistance, and other unfavorable prognostic outcomes (Shaukat et al., 2021). Hence, there is no disputing the urgency of improving therapeutic methods.

Shikonin is a natural compound derived from the root of *Lithospermum erythrorhizon*. Numerous studies have demonstrated that shikonin displays anticancer properties against various types of cancers, indicating its promise as a natural anticancer agent (Guo et al., 2019; Yadav et al., 2022). Previous studies have verified some of the possible mechanisms of shikonin in colon cancer. For instance, shikonin stimulates apoptosis and autophagy in colon cancer cells by targeting the *miR-545-3p/GNB1* signaling axis (Hu et al., 2022). In addition, shikonin exerts cytotoxic effects in colon cancer by inducing apoptosis via the endoplasmic reticulum and mitochondria-mediated pathways (Han et al., 2019). In addition, shikonin restrains colon cancer cell proliferation by inhibiting the *mTOR* pathway (Li et al., 2017). Meanwhile, shikonin may inhibit colon cancer cell growth by concurrently suppressing *ADAM17* and *IL-6/STAT3* signaling (Shi et al., 2021). It is well known that in addition to inhibiting cancer cell proliferation and promoting autophagy and apoptosis, shikonin may also inhibit cancer progression by inducing cancer cell senescence (Liu et al., 2020). Interestingly, other studies found that shikonin could inhibit lung cancer progression by promoting lung cancer cell senescence (Yeh et al., 2015; Zheng et al., 2018). Although various studies have investigated the role of shikonin in colon cancer, the underlying molecular mechanisms still need to be further explored.

Cellular senescence is a stable state of cell cycle arrest that is typically caused by injurious stimuli or pathological factors, including shortened telomeres, non-telomere DNA damage, and activation of oncogenes (Munoz-Espin and Serrano, 2014). It can prevent the formation of malignant tumors and hinder tumor progression by facilitating tumor cell senescence during cancer treatment (Calcinotto et al., 2019; Prasanna et al., 2021; Schmitt et al., 2022). In recent years, the extensive exploration of natural products in suppressing cancer progression through the promotion of cell senescence has garnered considerable interest among researchers, sparking a fascinating area of investigation. Some natural products have also been proven to inhibit tumor progression by promoting cell senescence in colon cancer. For example, *cucurbitacin* regulates the *miR-371b-5p/TFAP4* axis, causes senescence, and inhibits colon cancer progression in colon cancer cells (Yang et al., 2022). Likewise, *baicalin* induces colon cancer cellular senescence by upregulating *DEPP* and activating *Ras/Raf/MEK/ERK* signaling (Wang Z. et al., 2018). Nevertheless, whether shikonin promotes colon cancer cell senescence is unclear, and the mechanism by which shikonin promotes senescence is also not well-understood.

In our study, we investigated the inhibitory effects of shikonin on colon cancer cell lines HT29 and HCT116 and explored the pro-senescence molecular mechanism. The results showed that shikonin inhibits proliferation and migration of colon cancer cells by promoting cell senescence. Furthermore, the possible key genes *CDKN2A* and *CXCL8* were identified by systematic bioinformatics analysis and RT-qPCR. Based on our observations, we hypothesize that shikonin might play a pivotal role in inducing cellular senescence in colon cancer cells by suppressing the expression of CXCL8 and CDKN2A, ultimately leading to the inhibition of colon cancer development.

## 2 Materials and methods

### 2.1 Cell culture

The human colon cancer cell lines HT29 and HCT116 were purchased from the Cell Bank of the Chinese Academy of Sciences (Shanghai, China). The cells were cultured in DMEM (ExCell Biotech, Jiangsu, China) supplemented with 10% fetal bovine serum (FBS) (Invitrogen, Waltham, MA, United States) and 1% penicillin–streptomycin solution (Sangon Biotech, E607011, Shanghai, China). The cell incubator environment was 37°C, 5% CO_2_, and 95% O_2_. PBS was purchased from Sangon Biotech (E607008, Shanghai, China). Trypsin was also purchased from Sangon Biotech (E607002, Shanghai, China).

### 2.2 Drug

Shikonin (MB7082; purity >98%; Meilunbio, China) was dissolved in DMSO to form a storage solution with a concentration of 50 nM and stored at −20°C. The desired volume of the storage solution was removed for use and diluted to the appropriate concentration with cell culture, through which the final concentration of DMSO was 0.1%.

### 2.3 Cell proliferation assay

A total of 1.0 × 10^4^ cells were seeded in each well of 96-well plates and then incubated for 24 h. Next, the cells were treated with shikonin (0 μM, 3.93 μM for HCT116 and 0 μM, and 8.61 μM for HT29) for 24, 48, 72, 96, and 120 h, respectively. Cells were cultured in a solution containing 10% cell counting kit-8 (CCK-8, E1CK- 000208, Enogene) for 1 h in a cell incubator at 37°C. The absorbance was carefully measured at a wavelength of 450 nm.

### 2.4 Colony formation assay

A total of 1.0 × 10^3^ cells were seeded in each well of a 6-well plate. Following 24 h incubation, the cells were exposed to shikonin at concentrations of 0 μM and 3.93 μM for HCT116 and 0 μM and 8.61 μM for HT29, and they were maintained under these conditions for 14 days. Colonies were counted by crystal violet staining.

### 2.5 Wound healing assay

Each well of a 6-well plate was inoculated with 3.0 × 10^5^ cells. After 24 h, a 10-μL pipette tip was utilized to draw a vertical demarcation line at the center. Shikonin was then diluted with serum-free DMEM to the corresponding concentrations (0 μM and 3.93 μM for HCT116; 0 μM and 8.61 μM for HT29) and incubated for 24 h. The cells were then photographed under an optical microscope.

### 2.6 RNA isolation

HCT116 cells were cultured in 6-well plates at 3 × 10^5^ cells/well for 24 h. Cells were collected after 24 h of culturing with 0 μM and 3.93 μM shikonin, and total RNA was harvested by the Total RNA Extractor (TRIzol) reagent (Sangon Biotech, B511311, Shanghai, China). The specific steps were carried out according to the manufacturer’s instructions of TRIzol reagent.

### 2.7 mRNA high-throughput sequencing

NanoDrop 2000 (Thermo Fisher Scientific) was utilized to determine the RNA concentration and quality. Paired-end sequencing was performed on Illumina HiSeq2500. It was compared with the human reference genome hg38 using Tophat2 (http://ccbjhuedu/software/tophat) to analyze RNA-seq. featureCounts (http://subreadsourceforgenet) was used to calculated transcription read piece counts. DESeq2 (version 3.12) in R-studio version 4.0 was used for differentially expressed gene (DEG) analysis (fold change> 1.5, *p* < 0.05). Heat maps were created using ComplexHeatmap (Bioconductor project).

### 2.8 RT-qPCR

After 24 h treatment with shikonin (0 μM and 3.93 μM for HCT116; 0 μM and 8.61 μM for HT29), total RNA was harvested with TRIzol (Sangon Biotech, B511311, Shanghai, China). The concentration and purity of the RNA were determined and reverse-transcribed using a cDNA synthesis kit (TaKaRa Biotech, RR047A, Beijing, China) after the concentration and purity were determined. cDNAs were used as a template for real-time quantitative PCR (RT-qPCR) using FastStart Essential DNA Green Master Mix (Roche, 06924204001). All steps were performed according to the manufacturer’s procedures. Gene expression was normalized using the 2^−ΔΔCT^ method. The primers used for RT-qPCR are listed in Supplementary Table S1.

### 2.9 Senescence-associated β-galactosidase (SA-β-Gal) staining

Cells were treated with shikonin for 24 h (0 μM, 1.97 μM, and 3.93 μM for HCT116; 0 μM, 4.31 μM, and 8.61 μM for HT29); then, the medium was discarded gently, and the cells were rinsed once in phosphate-buffered saline (PBS). They were fixed with 2% formaldehyde for 3–5 min, and the formaldehyde was discarded; and they were rinsed three times with PBS for 3 min each. X-Gal solution (1 mg/mL) was added to each well, and the cell slides were immersed and incubated overnight at 37°C, as well as sealed through sealing film. The cells were then observed under an optical microscope and photographed.

X-Gal (Beyotime Biotechnology, ST912) was dissolved in DMSO and stored at −20°C in the dark as storage solution (200 mg/mL). The storage solution was diluted with NaCl (0.9%) to a final concentration of 1 mg/mL and then used.

### 2.10 Molecular docking

The receptor protein 3D structure (PDB code: 5WDZ) was obtained from the RCSB Protein Data Bank (http://www.rcsb.org/) (Wu et al., 2022). Molecular docking analysis was performed using Discovery Studio 2021 software. Shikonin was prepared using the “Prepare Ligands” module, and four conformations were generated by minimizing the CHARM force field. Next, the protein was prepared by hydrogenating and removing unnecessary water using the “Protein Preparation” module, and then, the protein was optimized and minimized. Finally, the ligand-binding site was verified as in a previous study (Cao et al., 2022). After performing CDOCKER docking using the “Receptor–Ligand Pharmacophore Generation” module, we used the hydrogen bond interactions and binding modes to evaluate the docking results. PyMol 3.7 was used to generate the 3D diagrams.

### 2.11 Statistical analysis

ImageJ and GraphPad Prism 5.0 were utilized for creating images and conducting data analysis, respectively. To compare the two groups' data, we employed the t-test. All data on t-test analysis meet the assumption of homogeneity of variances and normal distribution and are presented as mean ± standard error of mean (SEM). *P*-values < 0.05 were considered statistically significant.

## 3 Results

### 3.1 Shikonin inhibits the proliferation and migration of colon cancer cells

**FIGURE 1:**
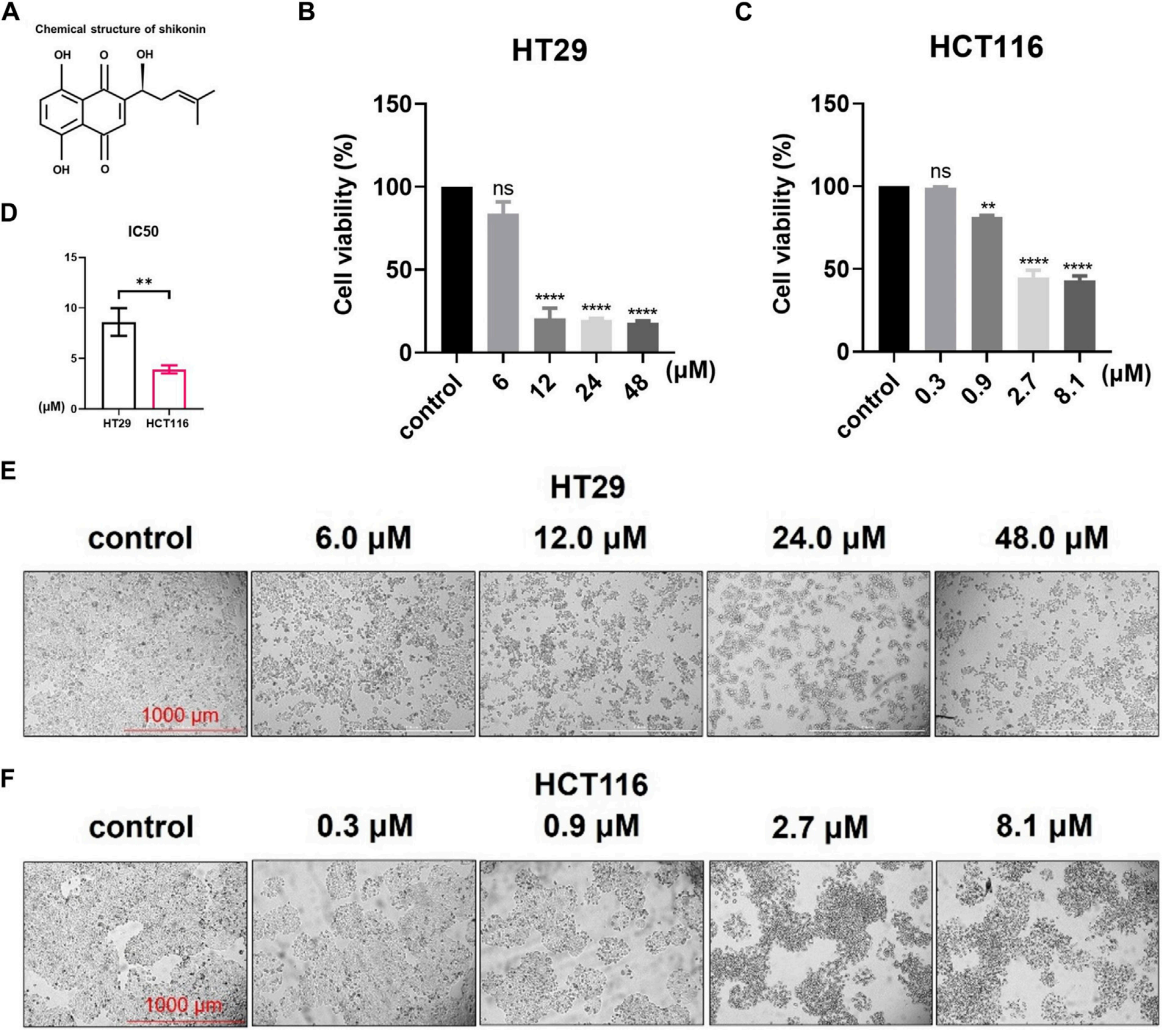
Shikonin treatment can inhibit colon cancer cell growth in a dose-dependent way. **(A)** Chemical structural formula of shikonin. **(B–D)** CCK-8 assay detecting the cell viabilities and IC_50_ values of HT29 and HCT116 cell lines. **(E,F)** Images of shikonin treatment dose-dependently inhibiting the growth of HT29 and HCT116 cells. Data are shown as mean ± S.E.M. **p < 0.001; ****p < 0.0001*.* Each experiment was performed in triplicate and repeated three times.

The structure of shikonin is shown in Figure 1A. Cell viability was detected using the CCK-8 assay (Figures 1B, C), and the IC_50_ values of shikonin in colon cancer cell lines HT29 and HCT116 were calculated as 8.61 μM and 3.93 μM, respectively (Figure 1D). As shown in the result, shikonin treatment suppressed cell growth in a dose-dependent manner in both HT29 and HCT116 colon cancer cell lines (Figures 1E, F).

**FIGURE 2:**
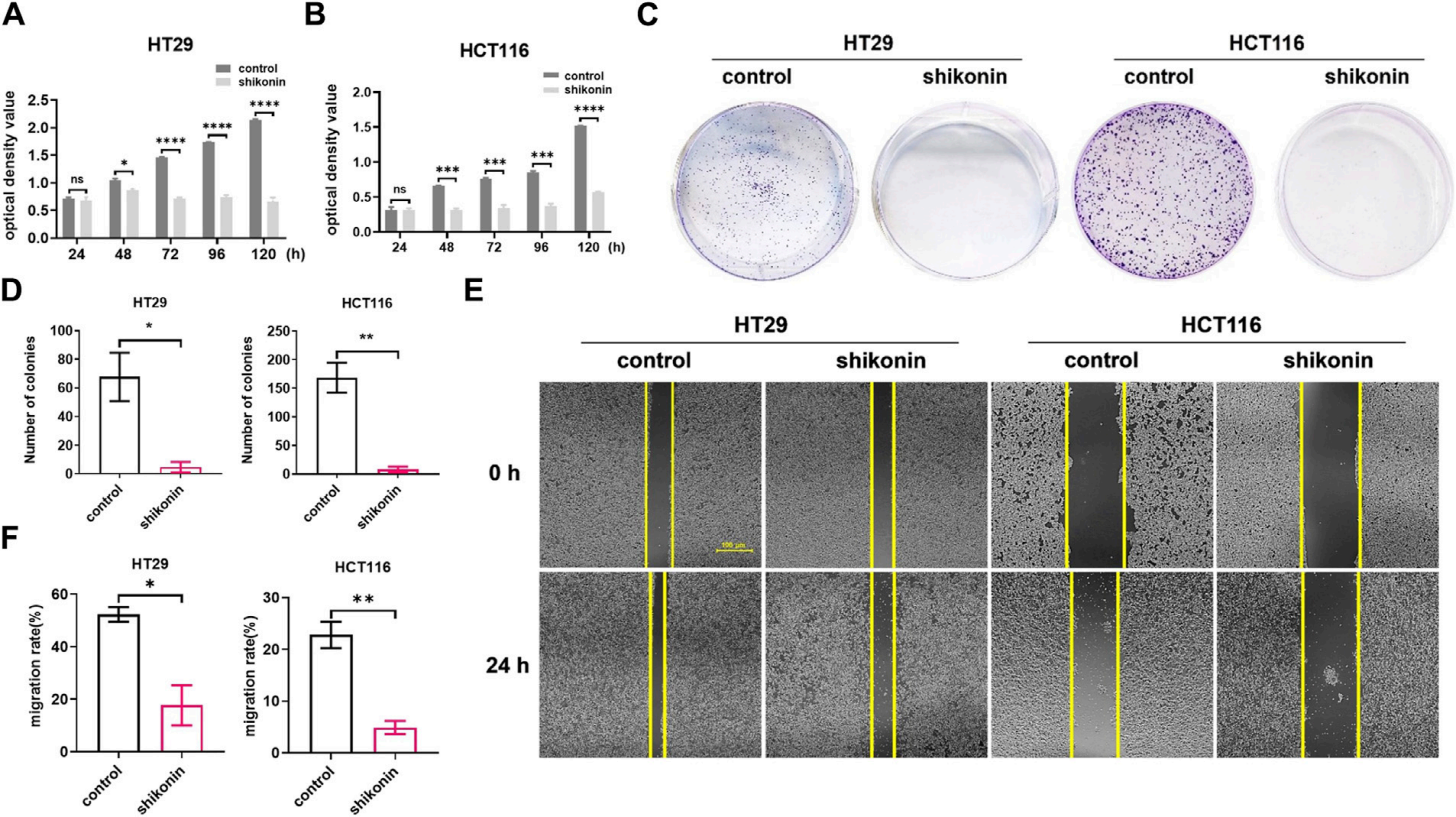
Shikonin treatment can inhibit the proliferation and migration abilities of colon cancer cells. **(A,B)** The proliferation abilities of HT29 and HCT116 colon cancer cells were inhibited after shikonin treatment by CCK-8 assay. **(C,D)** The proliferation abilities of HT29 and HCT116 colon cancer cells were inhibited after shikonin treatment by colony formation assay. **(E,F)** Wound healing assay determined that the migration abilities of HT29 and HCT116 cells are significantly decreased after shikonin treatment. Data are shown as mean ± S.E.M. *p < 0.05, **p < 0.001, ***p < 0.001, and ****p < 0.0001*.* Each experiment was performed in triplicates and repeated three times.

Next, we determined the inhibitory function of shikonin on colon cancer cell proliferation by cell proliferation assay and colony formation assay. Compared with the control group, the viability of both HT29 and HCT116 cells cultured with shikonin was significantly decreased (Figures 2A, B). Meanwhile, there are significantly more cell colonies formed in the control groups than in the groups treated with shikonin (Figures 2C, D, *p* = 0.00218 and *p* = 0.0038, respectively). The mean number of cell colonies was 68 in the HT29 control group and 5 in the HT29 shikonin treatment group. The average number of cell colonies was 169 in the HCT116 control group and 8 in the HCT116 shikonin treatment group (Figures 2C, D). These results indicate that shikonin exerted a profound inhibitory effect on the proliferation and growth of colon cancer cells.

To determine the role of shikonin in HT29 and HCT116 colon cancer cell migration, a wound healing assay was carried out. The cell migration rates of the shikonin treatment groups were significantly inhibited than those of the corresponding control groups (Figures 2E, F). The calculated migration rates for HT29 and HCT116 cells with shikonin treatment were 17.66% and 4.89%, respectively, which were significantly lower than those for the HT29 and HCT116 control groups, which were 52.30% and 22.81%, respectively (Figures 2E, F *p* = 0.0133 and *p* = 0.0032, respectively), indicating that shikonin had an inhibitory effect on the migration ability of colon cancer cells.

### 3.2 Searching for differentially expressed genes related to cellular senescence through mRNA sequencing and KEGG enrichment analysis

**FIGURE 3:**
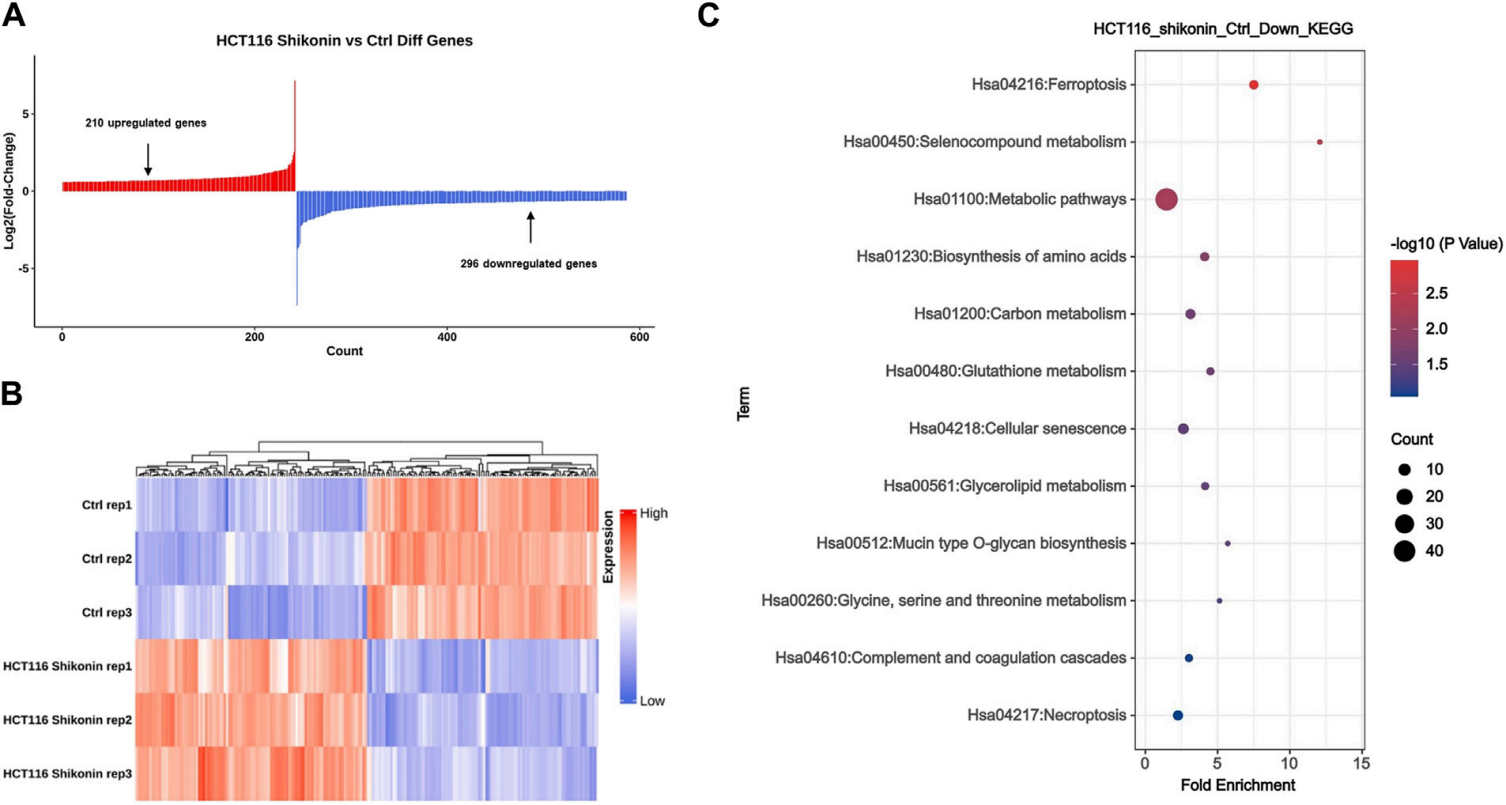
Shikonin may inhibit colon cancer cell progression through cellular senescence by comprehensive bioinformatics analysis. **(A)** mRNA sequencing analysis revealed a total of 210 upregulated genes and 296 downregulated genes after shikonin treatment. **(B)** Heatmap of the top 100 altered genes by shikonin treatment. **(C)** Kyoto Encyclopedia of Genes and Genomes (KEGG) enrichment analysis identified eight genes involved in cellular senescence, including *CDKN2A* and *CXCL8*.

The HCT116 cells from the treatment and control groups were collected for high-throughput mRNA sequencing. Comprehensive bioinformatics analysis showed that compared with the control colon cancer cells, 210 genes were upregulated and 296 genes were downregulated in colon cancer cells cultured with shikonin (Figure 3A). The top 100 altered genes are shown in Figure 3B. Through KEGG enrichment analysis, we discovered that a total of eight downregulated genes (*CCNB3, IL-1α, CXCL8, CDKN2A, MYC, IGFBP3, SQSTM1*, and *GADD45G*) were probably involved in inducing cell senescence (Figure 3C). *P16* ^*INK4a*^ and *P14* ^*ARF*^ (*P19* ^*ARF*^ in mice), encoded by *CDKN2A*, are critical genes involved in cellular senescence (Matheu et al., 2007; Serrano and Blasco, 2007; Baker et al., 2008). Other studies found that *CXCL8* is closely associated with the progression of colon cancer (Chen et al., 2019; Fang et al., 2022; Liu et al., 2022; Olivera et al., 2022), and the inhibition of *P16* ^*INK4a*^ expression also reduced the expression of *CXCL8* (Buj et al., 2021). Based on these observations, we hypothesized that *CXCL8* and *CDKN2A* are key regulators of cell senescence in HT29 and HCT116 colon cancer cells by shikonin.

**FIGURE 4:**
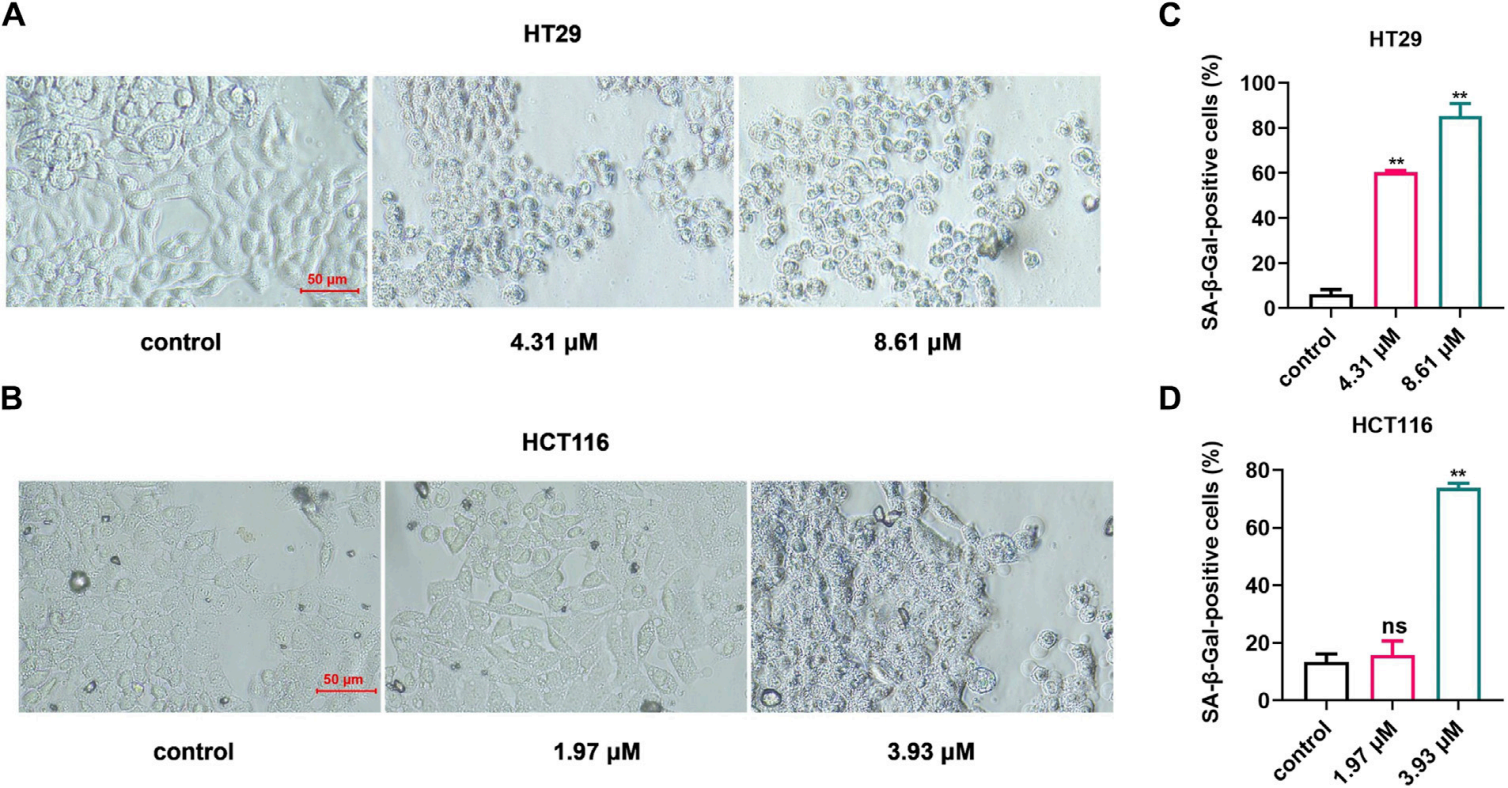
The proportion of senescent cells in colon cancer cells increased significantly after shikonin treatment. **(A,B)** Results of the senescence-associated β-galactosidase staining experiment in HT29 and HCT116 cells. **(C,D)** Calculation results of senescence-associated β-galactosidase staining experiment in HT29 and HCT116 cells. Data are shown as mean ± S.E.M. **p < 0.001. Each experiment was performed in triplicates and repeated three times.

To verify whether shikonin should potentially induce cell senescence in colon cancer cells by downregulating the expression of *CDKN2A* and *CXCL8*, we used a SA-β-Gal staining experiment to detect the senescent cells in the colon cancer cells that were treated with shikonin (Min et al., 2007; Hong et al., 2020). The results are shown in Figures 4A, B. Compared with the control group, the proportion of senescent cells in the shikonin treatment group was observably increased (Figures 4C, D; *p* = 0.0016 and *p* = 0.0059 in HT29 and *p* = 0.7014 and *p* = 0.0027 in HCT116), suggesting that shikonin induces colon cancer cell senescence, thereby inhibiting the development of colon cancer.

### 3.3 Shikonin may block colon cancer cell progression by downregulating the expression of *CDKN2A* and *CXCL8*

**FIGURE 5:**
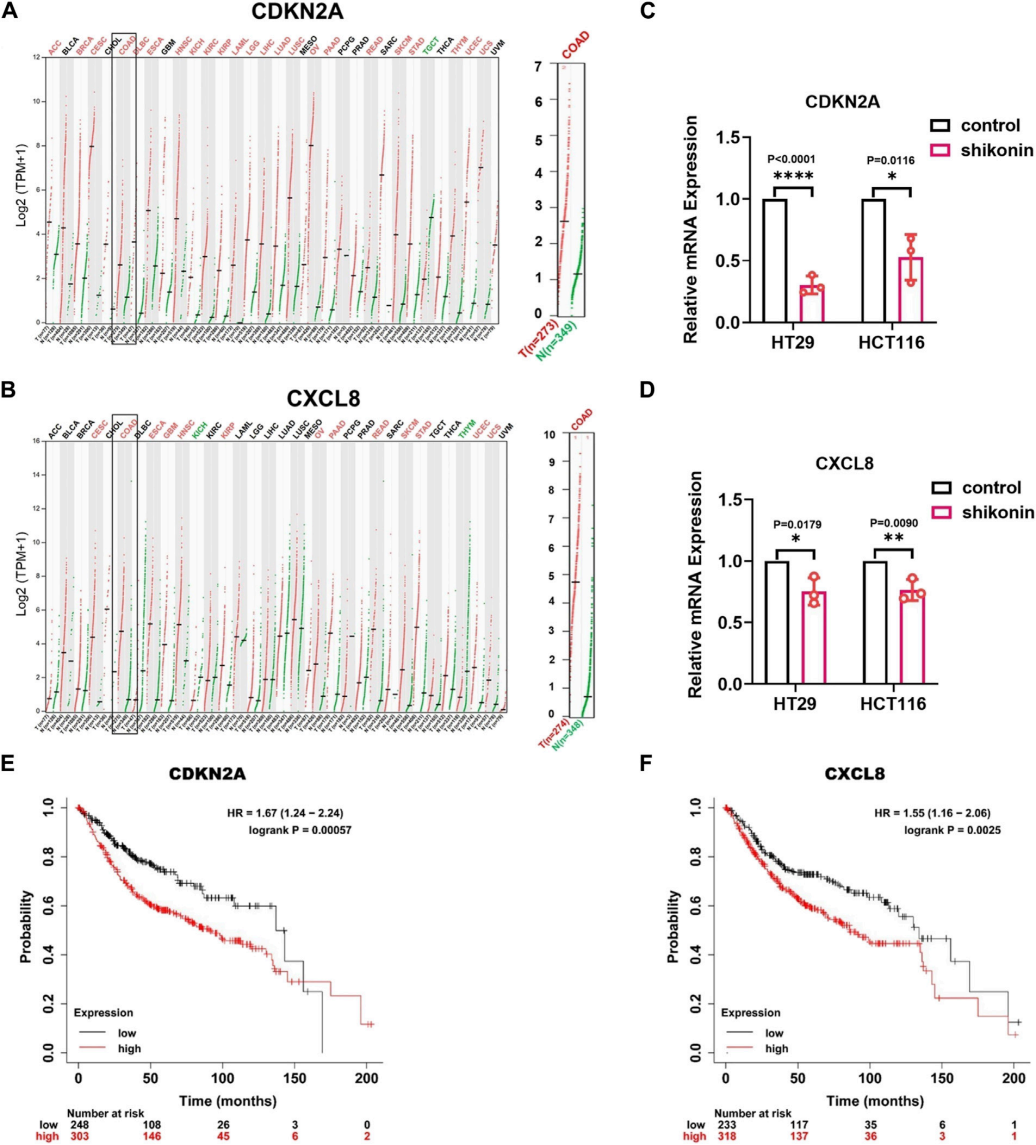
The expression of *CDKN2A* and *CXCL8* is downregulated in various cancers, and shikonin treatment can inhibit their expression levels in colon cancer cells. **(A,B)** Pan-cancer gene expression profiling of *CDKN2A* and *CXCL8*. **(C,D)** qPCR detection of *CDKN2A* and *CXCL8* in HT29 and HCT116. **(E,F)** Prognostic analysis showing the expression of *CDKN2A* and *CXCL8* negatively correlated with patient survival rates. Data are shown as mean ± S.E.M. *p < 0.05, **p < 0.001, and ****p < 0.0001. Each experiment was performed in triplicates and repeated three times.

Furthermore, to verify the mRNA expression levels of *CDKN2A* and *CXCL8* in colon cancer, we analyzed the pan-cancer gene expression levels using the GEPIA 2 database (http://gepia2.cancer-pku.cn/#index). *CDKN2A* and *CXCL8* were highly expressed in colon cancer and various other tumors (Figures 5A, B). Moreover, RT-qPCR experiments showed that after treatment with shikonin, the mRNA expression level of *CDKN2A* was downregulated 0.304 times in HT29 cells, 0.528 times in HCT116 cells (*p* < 0.0001 and *p* = 0.0116, respectively) and *CXCL8*, 0.752 times in HT29 cells, and 0.764 times in HCT116 cells (*p* = 0.0179 and *p* = 0.0090, respectively) (Figures 5C, D), which is consistent with our mRNA sequencing results. Furthermore, we wondered whether the low expression of *CDKN2A* and *CXCL8* following shikonin treatment was correlated with patient prognosis. Kaplan–Meier survival analysis was performed using data from the Kaplan–Meier plotter (http://kmplot.com/analysis/index.php?p=service). The results showed that patients with a high expression of *CDKN2A* and *CXCL8* had significantly lower survival rates than those with a low expression, indicating that *CXCL8* and *CDKN2A* were significantly correlated with poor prognosis in colon cancer (*p* < 0.005) (Figures 5E, F *p* = 0.00057 and *p* = 0.0025, respectively). Collectively, these results suggest that shikonin inhibits colon cancer development by downregulating the expression of *CDKN2A* and *CXCL8*, which may be involved in cellular senescence.

### 3.4 Interaction between shikonin and *CXCL8*

**FIGURE 6:**
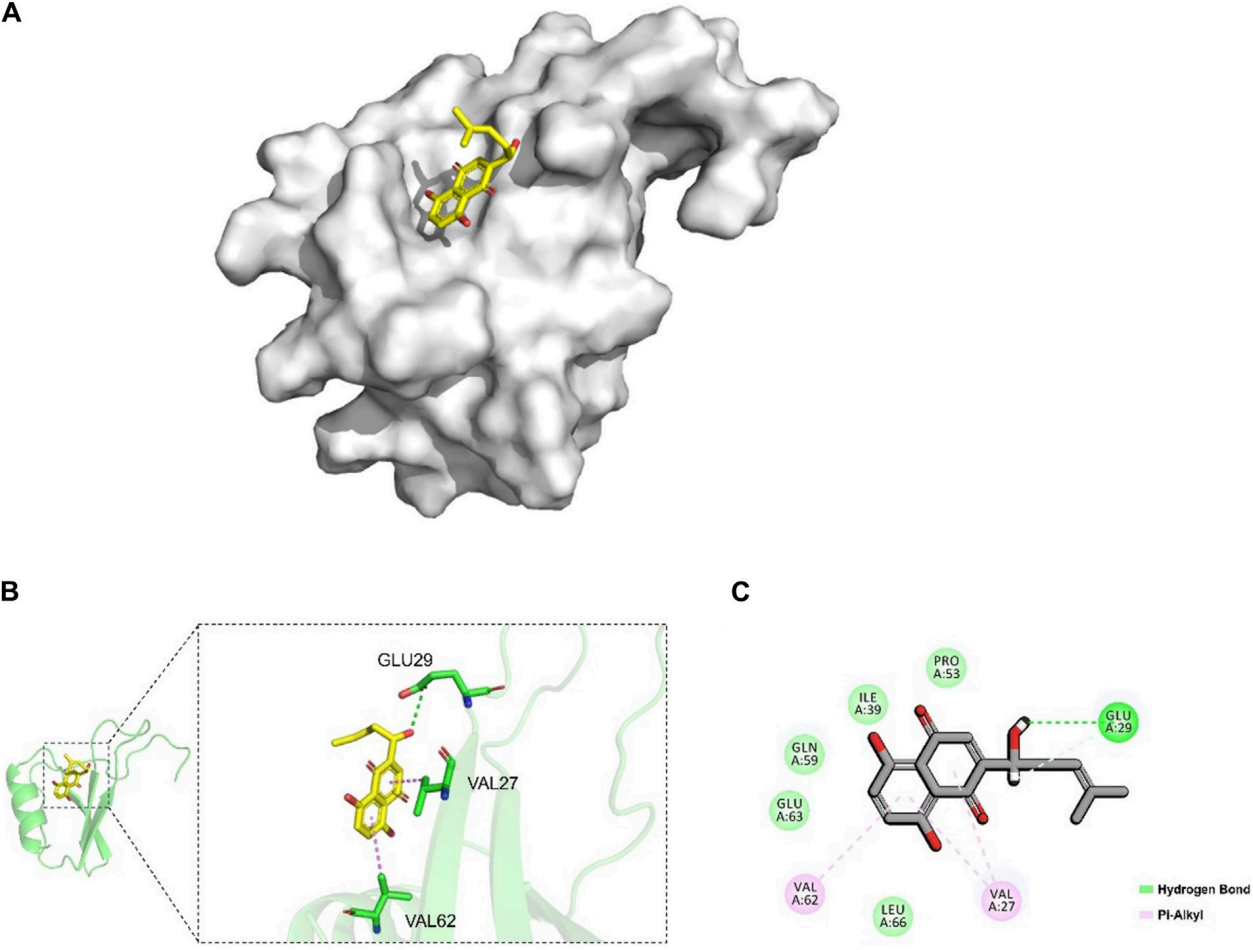
Molecular docking between shikonin and *CXCL8*. **(A)** Binding model of the shikonin (yellow) in the active pocket of *CXCL8* (gray). **(B)** Interaction of shikonin (yellow) with Glu29, Val27, and Val62 (green) of *CXCL8*. **(C)** 2D diagram of the interaction between shikonin and *CXCL8*. The hydrogen bond was represented by the dotted green line, and the hydrophobic bonds were represented by the dotted purple lines. Glu, glutamic acid; Val, valine.

To investigate whether there was an interaction between shikonin and *CXCL8*, we performed a molecular docking analysis, as shown in Figure 6A. The hydroxyl group of shikonin formed hydrogen bonds with the carbonyl group of Glu 29 of *CXCL8* (Figures 6B, C). In addition, we found that shikonin formed a hydrophobic interaction with Val 62 and Val 27 of *CXCL8*, supporting that shikonin could stably occupy the active pocket of *CXCL8* (Figures 6B, C). These results suggest that shikonin interacts with *CXCL8* and may inhibit the development of colon cancer by influencing *CXCL8* activity.

## 4 Discussion

Colon cancer has one of the highest incidences of malignant tumors threatening human health. The lack of timely screening, poor treatment effect, and short survival time of patients make it urgent to find new treatment methods (DeDecker et al., 2021; Shaukat et al., 2021). Compared with other anticancer drugs, natural drugs have significant advantages of declined toxicity and low cost (Liu et al., 2020). Current research has revealed that numerous natural products, including cucurbitin, baicalin, curcumin, and shikonin, exhibit significant inhibitory effects on colon cancer (Wang Z. et al., 2018; Fan et al., 2020; Hu et al., 2022; Yang et al., 2022). In our study, we found that shikonin effectively suppresses the proliferation and migration of colon cancer cells and identified that the potential mechanism underlying shikonin’s anticancer activity lies in its ability to induce cellular senescence.

Shikonin, as a natural naphthoquinone compound, possesses antitumor and anti-inflammatory properties (Yadav et al., 2022). In recent years, there has been a surge of research exploring the antitumor activity of shikonin. For example, shikonin inhibits the glycolysis of NSCLC cells and sensitizes cisplatin therapy through the exosome pyruvate kinase *M2* pathway (Dai et al., 2022). Likewise, shikonin inhibits the development of triple-negative breast cancer by inhibiting *IMPDH2* expression (Wang et al., 2021). Meanwhile, it has been confirmed that the *PKM2* inhibitor shikonin can improve the resistance of tumor cells to cisplatin during bladder cancer treatment (Wang Y. et al., 2018). To investigate the role of shikonin in the progression of colon cancer, we first verified that shikonin inhibited the proliferation, growth, and migration of HT29 and HCT116 colon cancer cells (Figures 1, 2). Current studies have shown that shikonin can inhibit the progression of colon cancer by inducing apoptosis and autophagy, enhancing NK cell proliferation and cytotoxicity, and inhibiting cell proliferation (Li et al., 2017; Liang et al., 2017; Han et al., 2019; Chen et al., 2021; Shi et al., 2021; Hu et al., 2022). In addition, previous research studies confirmed that shikonin inhibits lung cancer progression mainly by promoting cell senescence (Yeh et al., 2015; Zheng et al., 2018). However, it remains unclear whether shikonin suppresses colon cancer by inducing cellular senescence.

Cell senescence effectively halts the unchecked proliferation of cells that could lead to malignancy (Munoz-Espin and Serrano, 2014). In the present study, the KEGG analysis indicated that eight downregulated genes like *CXCL8* and *CDKN2A* were significantly associated with cell senescence (Figure 3) after shikonin treatment. As colon cancer remains a leading cause of cancer-related deaths globally, identifying such biomarkers is crucial for developing more effective treatment strategies. Similarly, several studies have indicated that *CXCL8* is related to tumor migration and invasion. High expression of *CXCL8* promotes tumor development, while inhibition of *CXCL8* expression can effectively inhibit tumor progression (Ha et al., 2017; Ogawa et al., 2019; Nie et al., 2021; Fu et al., 2023). Therefore, we postulate that shikonin, through its ability to induce senescence in colon cancer cells, may effectively downregulate the expression of *CXCL8* and *CDKN2A*. This process could potentially contribute to the inhibition of colon cancer development, offering a novel therapeutic approach.

Despite shikonin having demonstrated a remarkable inhibitory effect on colon cancer *in vivo* (Li et al., 2017; Liang et al., 2017; Chen et al., 2021; Hu et al., 2022), the question remains whether it can indeed exert its pro-senescence effect. On the other hand, while shikonin has been shown to have no systemic toxicities in tumor xenograft mice, it is essential to conduct further studies to ensure there are no other potential *in vivo* toxic effects. This is crucial for ensuring the safety of shikonin as a potential therapeutic agent (Zheng et al., 2018). Current research efforts should be focused on improving the structure of shikonin and verifying the effectiveness of its less toxic derivatives in tumor therapy (Guo et al., 2019). Additionally, studies on dosage forms are aiming to enhance the bioavailability of shikonin, making it more effective and tolerable in clinical settings (Yan et al., 2023). Given the significant progress made in understanding the anticancer properties of shikonin, further studies on the molecular mechanisms underlying its anti-tumor effects, especially those related to inducing senescence, are eagerly anticipated.

## 5 Conclusion

**FIGURE 7:**
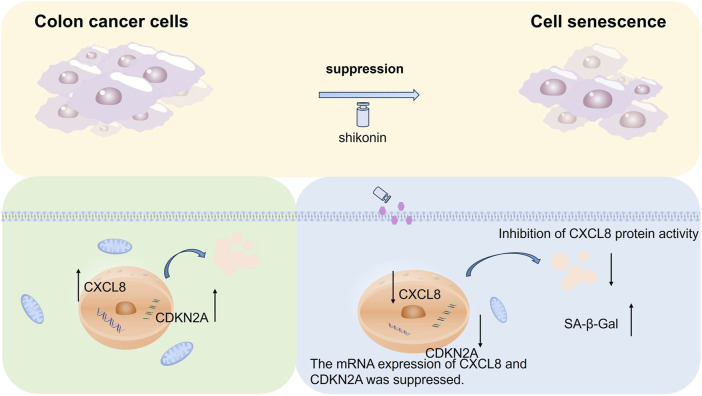
Diagram of the molecular mechanism for the present study. Shikonin may induce colon cancer cell senescence by downregulating *CDKN2A* and *CXCL8* and suppress colon cancer development.

In summary, we found that shikonin suppresses colon cancer cell proliferation and migration through cellular experiments and identified eight genes (*CCNB3, IL-1α, CXCL8, CDKN2A, MYC, IGFBP3, SQSTM1,* and *GADD45G*) associated with cell senescence through systematic bioinformatics analysis. The senescence-associated genes *CDKN2A* and *CXCL8* were significantly downregulated in colon cancer cells after shikonin treatment. We investigated the altered genes after shikonin treatment by mRNA sequencing and identified eight genes associated with cellular senescence in combination with Kyoto Encyclopedia of Genes and Genomes (KEGG) analysis. Among the downregulated genes, we selected *CDKN2A* and *CXCL8* as targets to further explore their roles in colon cancer progression. We verified that shikonin downregulates the mRNA expression of *CXCL8* and *CDKN2A* using quantitative polymerase chain reaction (qPCR). Additionally, prognostic analysis of the two target genes was performed to confirm that their downregulation correlated with poor prognosis in patients with colon cancer. We found that shikonin increased the activity of senescence-associated β-galactosidase (SA-β-Gal) in colon cancer cells after downregulation of *CXCL8* and *CDKN2A* through a senescence-associated β-galactosidase (SA-β-Gal) staining experiment. Molecular docking analysis revealed that shikonin promotes cellular senescence in colon cancer cells by blocking the activity of *CXCL8* at the protein level. Based on these findings, we propose that shikonin may induce senescence in colon cancer cells and inhibit colon cancer progression by downregulating *CDKN2A* and *CXCL8* (Figure 7).

## Data Availability

The original contributions presented in the study are publicly available. This data can be found here: https://www.ncbi.nlm.nih.gov/geo/query/acc.cgi?acc=GSE234709, accession number GSE234709.
